# Mosaic Evolution of Membrane Transporters in Galdieriales

**DOI:** 10.3390/plants14132043

**Published:** 2025-07-03

**Authors:** Claudia Ciniglia, Antonino Pollio, Elio Pozzuoli, Marzia Licata, Nunzia Nappi, Seth J. Davis, Manuela Iovinella

**Affiliations:** 1 Department of Environmental, Biological and Pharmaceutical Sciences and Technologies, University of Campania “L. Vanvitelli”, Via Vivaldi 43, 81100 Caserta, Italy; claudia.ciniglia@unicampania.it (C.C.); elio.pozzuoli@unicampania.it (E.P.); 2 Department of Biology, University of Naples Federico II, Via Cinthia, 80126 Napoli, Italy; anpollio@unina.it (A.P.); marzia.licata@unina.it (M.L.); nunzia.nappi@unina.it (N.N.); 3 Department of Biology, University of York, Wentworth Way, York YO10 5DD, UK

**Keywords:** Galdieriales, transmembrane transporters, REEs, phylogeny, HGT

## Abstract

Membrane transporters are vital for solute movement and localisation across cellular compartments, particularly in extremophilic organisms such as Galdieriales. These red algae thrive in geothermal and metal-rich environments, where adaptive transporter systems contribute to their metabolic flexibility. While inventories of transporter genes in the species *Galdieria sulphuraria* have previously been compiled, their phylogenetic origins remain incompletely resolved. Here, we conduct a comparative phylogenetic analysis of three transporter families—Major Facilitator Superfamily (MFS). Amino acid–Polyamine–Organocation (*APC*) and the natural resistance–associated macrophage protein (Nramp)—selected from overexpressed transcripts in *G. sulphuraria* strain SAG 107.79. Using sequences from six Galdieriales species and orthologs from diverse taxa, we reconstructed maximum likelihood trees to assess conservation and potential horizontal gene transfer (HGT). The MFS subfamilies revealed contrasting patterns: sugar porters (SPs) exhibited polyphyly and fungal affinity, suggesting multiple HGT events, while phosphate:H^+^ symporters (*PHS*s) formed a coherent monophyletic group. *APC* sequences were exclusive in *G. sulphuraria* and extremophilic prokaryotes, indicating a likely prokaryotic origin. In contrast, Nramp transporters were broadly conserved across eukaryotes and prokaryotes, showing no signs of recent HGT. Together, these findings highlight the mosaic evolutionary history of membrane transporters in Galdieriales, shaped by a combination of vertical inheritance and taxon-specific gene acquisition events, and provide new insight into the genomic strategies underpinning environmental resilience in red algae.

## 1. Introduction

Membrane transporters are essential proteins that mediate the movement and localisation of solutes across cellular compartments. These proteins support nutrient uptake, ion homeostasis, detoxification, and adaptation to extreme environments. Among the most represented in eukaryotic and prokaryotic genomes are members of the Major Facilitator Superfamily (MFS), the Amino Acid–Polyamine–Organocation (APC) family, and the natural resistance–associated macrophage protein (Nramp) metal ion transporters, members of the large APC superfamily [1,2,3].

The Major Facilitator Superfamily (MFS) is the largest and among the most extensively characterised protein families involved in solute transport across lipid bilayers. The reaction could involve two or more substrates, which can move in the same direction (MFS symporters) or in opposite one (MFS antiporters). Until now, the crystal structure of only seven *MFS* proteins, belonging to six subfamilies, was characterised [1]. Bioinformatic tools were also widely employed to predict the structure of these proteins. The predictions identified 12 or more transmembrane helices (TMS) [1], organised into two distinct and folded domains, the N and C domains, whose termini are located on the cytoplasmic side of the membrane [1]. This structure, called the MFS fold, is shared across all six subfamilies, even though the proteins widely differ in amino acid sequence, substrate specificities, and transport coupling mechanisms [1].

The amino Acid–Polyamine–Organocation (APC) family is the second largest superfamily of secondary carriers after the Major Facilitator Superfamily (MFS) and includes proteins acting as solute/cation symporters or solute/solute antiporters [2,4]. Some APC proteins facilitate the transmembrane movement of a wide range of amino acids or related compounds, while others permit the translocation of only a subset [2]. Farcasanu et al. in 1998 described the involvement of a high-affinity permease for histidine in the transport of Mn^2+^. The protein acquired its new functionality through a mutation in the coding gene, which resulted in a frameshift and protein truncation [5]. Collectively, the functional versatility within the APC family highlights its evolutionary adaptability in enabling organisms to thrive under diverse and challenging environmental conditions.

The family of natural resistance–associated macrophage protein (Nramp) metal ion transporters comprises essential proteins related to metal ion transport across cellular membranes and metal ion homeostasis [3,6], such as trace metals like Cu^2+^, Mn^2+^, Fe^2+^, and Zn^2+^. These are essential cofactors for many enzymes [6]. Nramp proteins also intervene when an excess of heavy metals, such as cadmium, lead, and mercury, occurs, extruding them through the plant’s root or sequestering them within cell compartments, such as the vacuole [3].

In *Galdieria sulphuraria*, an extremophilic red microalgae that thrives in geothermal and metal-rich environments [7,8,9,10], such transporters are presumed to play a central role in metal tolerance and metabolic versatility [11]. Early genomic work on *G. sulphuraria* and its relative *Cyanidioschyzon merolae* catalogued metal transporter families based on predicted homology, sequence domains, and subcellular localisation [12,13]. Comparative studies with *Chlamydomonas reinhardtii* and other algae revealed marked differences in transporter repertoires that reflect ecological specialisations. Subsequent inventories emphasised an enrichment of secondary carriers, particularly MFS and PHS transporters, in *G. sulphuraria* [11]. However, while transporter counts and functional predictions have been described, the phylogenetic placement of these genes across Galdieriales species and against wider taxa remains poorly resolved. Moreover, the evolutionary origin of these genes—some of which appear to have derived from Archaea, Bacteria, or Fungi—has only been inferred from broad, genome-wide surveys [12].

Here, we undertake a focused phylogenetic characterisation of selected Galdieriales genes encoding proteins related to membrane transport, as defined by the Gene Ontology category “Localization” (GO:0051179), including members of the MFS, APC, and Nramp families. Here, we used genomic data as a starting point for comparative analysis across Galdieriales species and a broad sampling of taxa to resolve phylogenetic relationships and potential horizontal gene transfer (HGT) events. This work updates and extends the inventory approach pioneered by Hanikenne et al. (2005), seeking to deepen our understanding of the evolution and diversification of transporter families in extremophilic red algae [13].

## 2. Results

### 2.1. Major Facilitator Superfamily (MFS) Relationships Reveal Evidence of Polyphyly and Fungal Affinity 

In silico analyses based on Phobius and Protter web server identified two different transmembrane domains that differ in the number of alpha-helix crossing the double layer of the membrane and the locations of the N- and C-termini. The Major Facilitator Superfamily (MFS), Sugar Porter (SP) subfamilies (hereinafter referred to as the MFS-SP subfamily) of *G. sulphuraria* ACUF 017, *G. javensis* ACUF 074, *G. yellowstonensis* SAG 107.79, *G. sulphuraria* ACUF 138, and *G. partita* THAL 033 consist of 12 transmembrane domains (TMDs), with the N- and C-termini located intracellularly (Box 1). The analyses also identified an N-glycosylation motif (green-labelled) at amino acid position 402, shared across all these species. *G. daedala* ACUF 427 prediction highlighted 11 TMDs and identified the N-terminus extracellularly and two N-glycosylation motifs at the amino acid positions 341 and 574 (Box 1).

Box 1 **Predicted transmembrane domains of the MFS-SP subfamily. TMHMM and PROTTER web software were used to predict the transmembrane domains and N- and C-termini.**

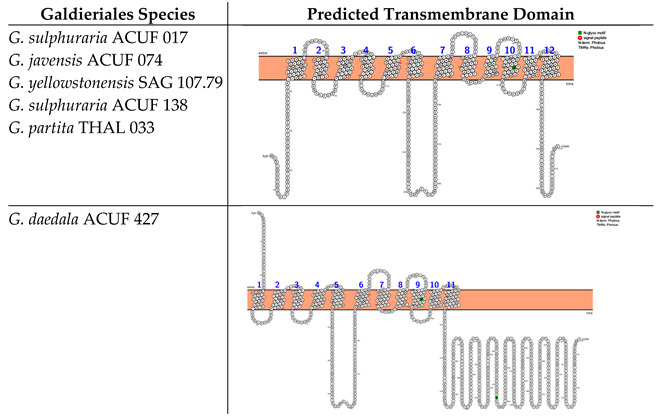

**Figure 1:**
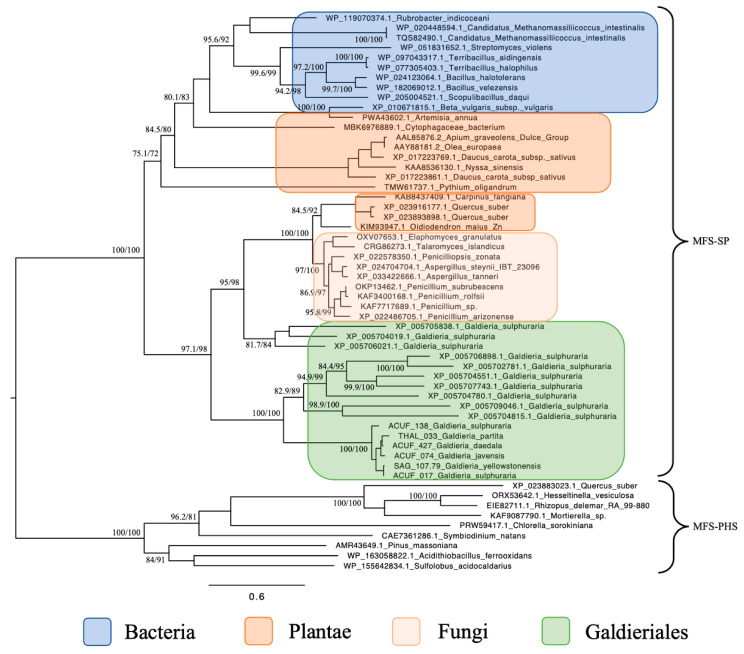
Maximum likelihood tree of the MFS-SP subfamily. Ultrafast bootstrap (UFBoot), the Approximate Likelihood Ratio Test (aLRT) and Shimodaira–Hasegawa (SH-aLRT) support values are indicated near the nodes. Alien index = 193.23; e-value = 1.2 × 10^−84^; donor taxonomy = Kingdom Fungi.

The maximum likelihood phylogenetic tree constructed for the MFS-SP subfamily revealed a complex and polyphyletic evolutionary pattern for these transporters across eukaryotes and extremophiles (Figure 1). Focusing on Galdieriales, sequences extracted from six genomes clustered together into a statistically robust monophyletic clade (100% UFBoot and 100% SH-aLRT), suggesting a shared evolutionary origin within the genus. Within this clade, substructure was evident. The closest relationship was observed between *G. yellowstonensis* SAG 107.79 and *G. sulphuraria* ACUF 017 (99% UFBoot and 94.6% SH-aLRT), which formed a sister pair. Similarly, *G. javensis* ACUF 074 and *G. daedala* ACUF 427 grouped tightly together (98% UFBoot and 78.9% SH-aLRT). *G. partita* THAL 033 showed moderate divergence from this latter pair (85% UFBoot and 67.6% SH-aLRT), while *G. sulphuraria* ACUF 138 formed a distinct and more deeply branching lineage within the clade (100% UFBoot and 100% SH-aLRT; Figure 1).

When placed in a broader phylogenetic context, the entire Galdieriales clade was found to form a sister group to a set of homologous sequences retrieved from the NCBI nr database, which were also annotated as *Galdieria* spp. transporters. This expanded Galdieriales cluster, incorporating both newly sequenced and public-domain data, was strongly supported as a clade (100% UFBoot and 100% SH-aLRT) and showed clear phylogenetic affinity with fungal sequences, indicating a likely horizontal gene transfer (HGT) event from fungi to the Galdieriales species. Interestingly, the broader fungal–Galdieriales clade also contained sequences from two higher plant species, *Carpinus fangiana* and *Quercus suber*, which clustered within the fungal group rather than forming a distinct plant outgroup. This unexpected placement suggested possible misannotation, deep paralogy, or rare gene exchange events and further supported the hypothesis of a polyphyletic origin for SP subfamily proteins. Additional Galdieriales sequences retrieved from NCBI were also scattered across this fungal-dominated clade, suggesting multiple independent acquisition events or functional diversification following HGT (Figure 1).

The prediction of the transmembrane domains for the Major Facilitator Superfamily (MFS), Phosphate:H^+^ Symporter (*PHS*) subfamily (hereinafter referred to the MFS-PHS subfamily) highlighted two different transmembrane domains, distinguished by the number of alpha-helices. The first transmembrane protein structure was identified in *G. sulphuraria* ACUF 017, *G. javensis* ACUF 074, *G. yellowstonensis* SAG 107.79, *G. sulphuraria* ACUF 138, and *G. partita* THAL 033 and consisted of 12 TMDs with both N- and C-termini located intracellularly. These proteins were characterised by three N-glycosylation motifs at amino acid positions 153, 314, and 455 (Box 2). The second kind of protein structure was identified in *G. daedala* ACUF 427, consisting of 10 TMDs and the N- and C-termini on the intracellular side of the membrane and characterised by the presence of three N-glycosylation motifs at different amino acid positions compared to the other species (35, 151, and 312; Box 2).

Box 2 **Predicted transmembrane domain of the MFS-PHS subfamily. TMHMM and PROTTER web software tools were used to predict the transmembrane domains and the N- and C-termini.**

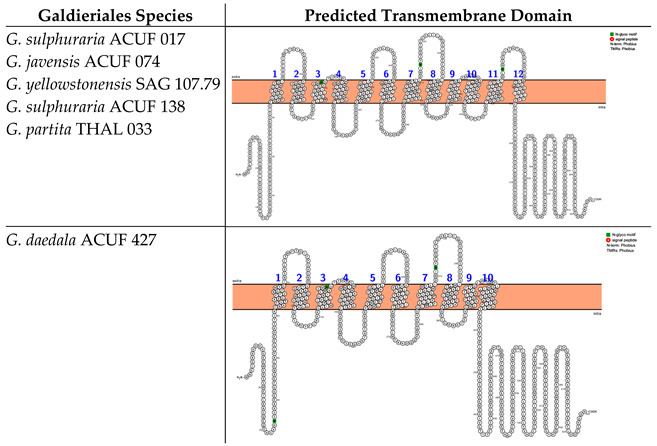

**Figure 2:**
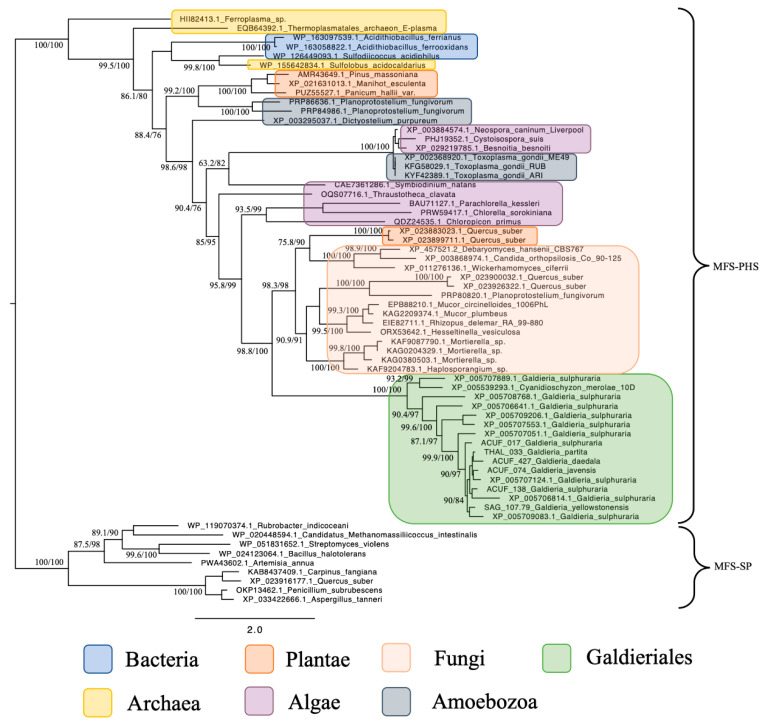
Maximum likelihood tree of the MFS-*PHS* subfamily. Ultrafast bootstrap (UFBoot), the Approximate Likelihood Ratio Test (aLRT), and Shimodaira–Hasegawa (SH-aLRT) support values are indicated near the nodes. Alien index = 113.56; e-value = 4.8 × 10^−50^; donor taxonomy = Kingdom Fungi.

Phylogenetic reconstruction of the Phosphate:H^+^ Symporter (*PHS*) subfamily, another branch of the MFS superfamily, revealed a much simpler and coherent monophyletic origin (Figure 2). All Galdieriales sequences clustered tightly into a single clade, again with maximal support (100% UFBoot and 100% SH-aLRT). This uniformity suggested a conserved functional lineage within Galdieriales and provided no evidence for duplication, subfunctionalisation, or polyphyly within the genus. As with the SP subfamily, the Galdieriales *PHS* clade grouped closely with fungal sequences. However, the fungal taxa involved were not the same as those observed in the SP phylogeny. This non-overlapping pattern of fungal associations between the two subfamilies suggests that independent HGT events may have introduced the SP and *PHS* transporters into the Galdieriales genome, likely originating from distinct fungal sources (Figure 2). Alternatively, divergent evolutionary pressures may have shaped these families post-transfer. Collectively, these data underscore the mosaic evolutionary history of MFS transporters in Galdieriales. The contrast between polyphyletic SP origins and monophyletic *PHS* inheritance, along with their divergent fungal affinities, highlighted the complexity of transporter gene evolution in extremophilic red algae and reinforced the notion that horizontal gene transfer has been a key driver of niche adaptation in this lineage.

### 2.2. Amino Acid–Polyamine–Organocation (APC) Family Sequences Clustered Exclusively with Extremophilic Prokaryotes

The analysis of transmembrane domains for the Amino Acid–Polyamine–Organocation family (hereinafter referred to as the APC-family) unveiled three distinct transmembrane configurations that vary in their number of alpha-helices and in the location of the N- and C-termini. The first category of protein structure was identified in *G. daedala* ACUF 427, *G. sulphuraria* ACUF 017, and *G. yellowstonensis* SAG 107.79. This structure was characterised by 12 TMDs, with both the N- and C-termini positioned intracellularly. Moreover, these proteins exhibited two N-glycosylation motifs at amino acid positions 82 and 403 (Box 3). The second protein category was found in *G. javensis* ACUF 074 and *G. partita* THAL 033, which features a streamlined configuration with 14 TMDs, also with both the N- and C-termini on the intracellular side of the membrane and two N-glycosylation motifs at the same position as the other species (82 and 403; Box 3).

Box 3 **Predicted transmembrane domain of the APC family. TMHMM and PROTTER web software were used to predict the transmembrane domains and the N- and C-termini.**

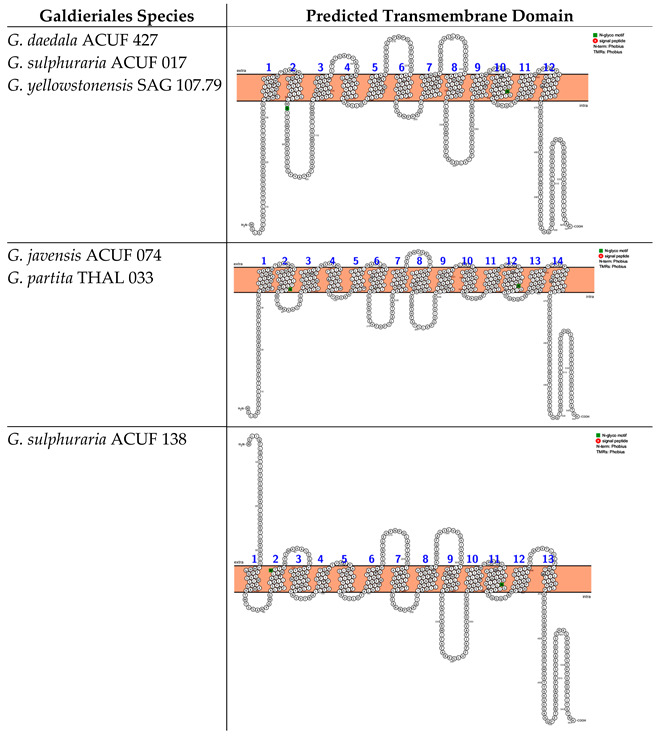

**Figure 3:**
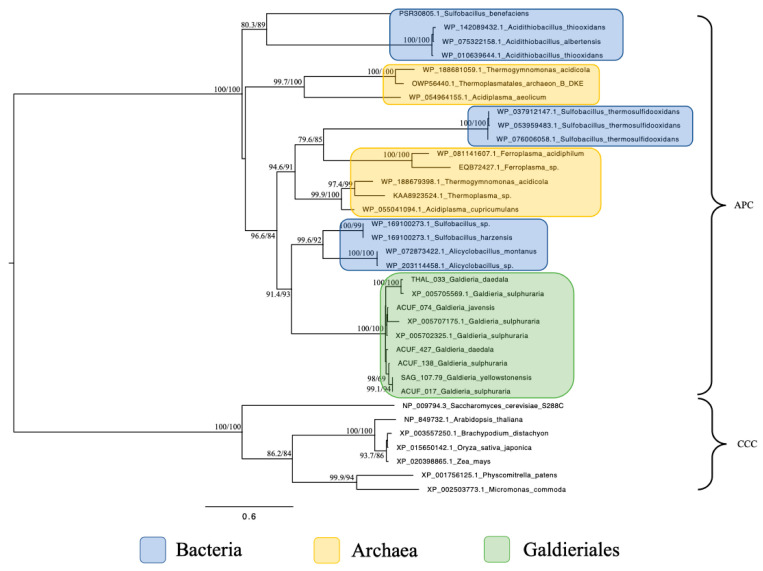
Maximum likelihood tree of the Amino Acid–Polyamine–Organocation (APC) family. Ultrafast bootstrap (UFBoot), the Approximate Likelihood Ratio Test (aLRT0 and Shimodaira–Hasegawa (SH-aLRT) support values are indicated near the nodes. Sequences from Plant Cation-Chloride Cotransporters (CCCs) were chosen as the outgroup [14]. Alien index = 232.60; e-value = 9.62 × 10^−102^; donor taxonomy = Kingdom Bacillati.

Phylogenetic analysis of the Amino Acid–Polyamine–Organocation (APC) family revealed a narrow distribution of homologues, confined to extremophilic prokaryotic taxa (Figure 3). All Galdieriales sequences formed a single, statistically well-supported monophyletic clade (100% UFBoot and 100% SH-aLRT). Internal variation among sequences from different Galdieriales species was low, with several strain-specific branches also supported (e.g., 98% UFBoot and 69% SH-aLRT). No eukaryotic sequences outside Galdieriales were retrieved for these APC sequences, distinguishing them from the other transporter families analysed in this study. The Galdieriales clade grouped with sequences from extremophilic Bacteria, with a strong support (93% UFBoot and 91.4% SH-aLRT). A broader clade including Galdieriales species and multiple Archaea showed high statistical support (84% UFBoot and 96.6% SH-aLRT; Figure 3). These results indicate that APC homologues in Galdieriales are conserved among species, not shared with other eukaryotes, and demonstrate a close phylogenetic relationship with extremophilic prokaryotes.

### 2.3. Nramp (Natural Resistance–Associated Macrophage Protein) Family, Associated with Mn^2+^ and Fe^2+^ Transport, Was Widely Distributed Across Eukaryotic and Prokaryotic Lineages

Examination of transmembrane domains within the Nramp (natural resistance–associated macrophage protein) family, associated with Mn^2+^ and Fe^2+^ transport, has revealed one unique transmembrane structure with low variability. The identified protein architecture, based on 12 transmembrane domains (TMDs) with both the N-terminus and C-terminus situated on the intracellular side, and the absence of N-glycosylation motifs, was shared across all the Galdieriales species (Box 4).

Box 4 **Predicted transmembrane domains of the Nramp family, associated with Mn2+ and Fe2+ transport. TMHMM and PROTTER web software were used to predict the transmembrane domain and the N- and C-termini**

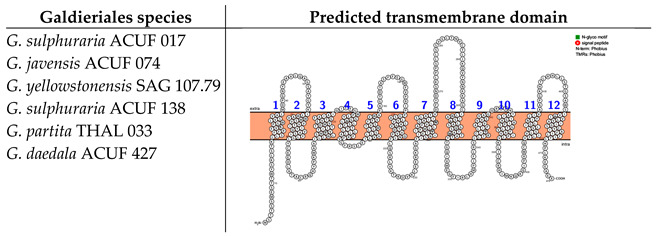

**Figure 4:**
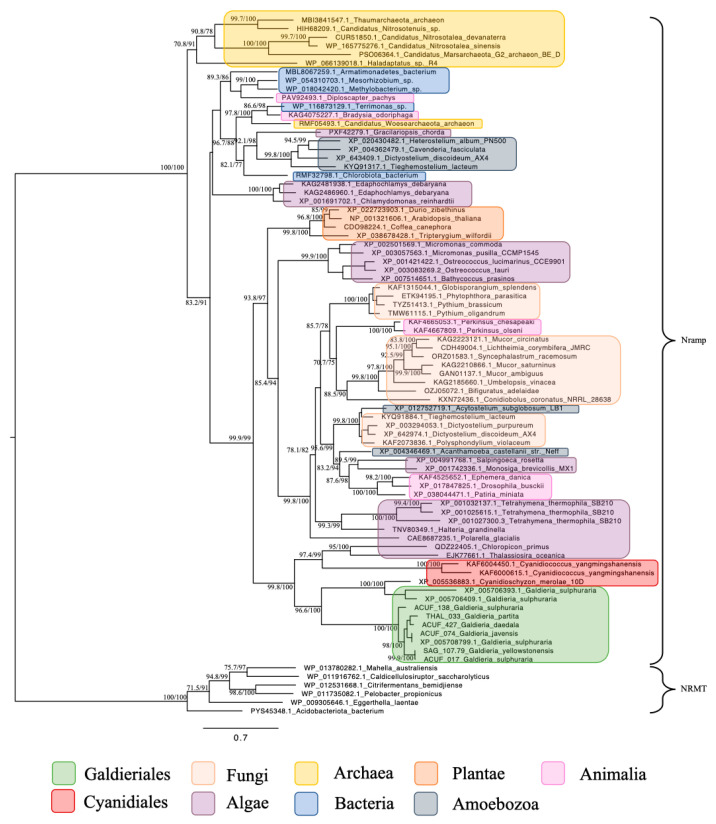
Maximum likelihood tree of the Metal ion (Mn^2+^/Fe^2+^) transporter, *Nramp* family. Ultrafast bootstrap (UFBoot), the Approximate Likelihood Ratio Test (aLRT), and Shimodaira–Hasegawa (SH-aLRT) support values are indicated near the nodes. Sequences from NRAMP- related magnesium transporters (NRMTs) were chosen as the outgroup [15].

For the subsequent phylogenetic analyses, genes encoding for the *Nramp* family, associated with Mn^2+^ and Fe^2+^ transport, were identified across a wide taxonomic range (Figure 4). Homologues were detected in both prokaryotic and eukaryotic domains, including Bacteria, Archaea, Fungi, Chlorophyta, Streptophyta, Stramenopiles, Alveolata, Amoebozoa, Choanoflagellates, and Animals. This widespread distribution underscores the evolutionary conservation and fundamental biological importance of *Nramp* transporters in diverse cellular contexts. Within Galdieriales, sequences formed two distinct branches: One branch contained all Galdieriales sequences newly generated and derived from transcriptomic data obtained from the species *G. yellowstonensis* treated with the rare earth element Cerium (25 mg/L). This clade was fully supported by phylogenetic statistics (100% UFBoot and 100% SH-aLRT), indicating high sequence conservation among the six Galdieriales species examined (Figure 4). The second adjacent branch comprised additional Galdieriales sequences retrieved from the NCBI database, along with sequences from two other Cyanidiales red algae: *Cyanidioschyzon merolae* and *Cyanidiococcus yangmingshanensis*. This clade was also fully supported (100% UFBoot and 100% SH-aLRT). The two Cyanidiales clades together formed a larger, well-supported group (100% UFBoot and 96.6% SH-aLRT), which was placed as a sister group to the algae clade with strong statistical support (100% UFBoot and 98.8% SH-aLRT). The broader tree topology indicated that, while *Nramp* homologues are taxonomically widespread, Galdieriales sequences formed a distinct and cohesive lineage within the family (Figure 4).

### 2.4. Protein Structure Prediction and Confidence Assessment

The 3D structures of the target proteins were predicted using ColabFold v1.5.5: AlphaFold2, yielding high-confidence. The pLDDT values mapped onto the structures revealed core regions with well-defined transmembrane core domains with pLDDT > 90. Only *NRamp* showed a lower pLDDT in the same region (Figures S1–S4).

## 3. Discussion

Galdieriales species have recently been exploited for the recovery of precious and rare earth metals, thanks to their impressive capability to tolerate high environmental metal concentrations [16,17,18,19,20,21]. These microalgae have also been shown to selectively absorb different rare earth metals, resolving one of the main issues when trying to recycle single rare earth elements from a mixture of metals [22].

This study provides a focused phylogenetic characterisation of membrane transporter families in Galdieriales, building on previous inventory-style surveys by exploring the evolutionary origins of key transcripts. Transcriptomic analyses of *G. yellowstonensis* SAG 107.79, treated with the rare earth element Cerium (25 mg/L) and collected after one hour of treatment revealed elevated expression of genes involved in the Gene Ontology categories “Localization” (GO:0051179) and “Transport” (GO:0006810), including representatives of the Major Facilitator Superfamily (MFS), the Amino Acid–Polyamine–Organocation (APC) family, and the *Nramp* family of metal ion transporters. While previous work inferred transporter abundance and diversity in *G. sulphuraria* [11,12], the present phylogenetic approach helps to resolve the taxonomic affinities and potential horizontal gene transfer (HGT) events associated with these families.

Earlier genomic analyses identified a total of 477 transporters in *G. sulphuraria*, with secondary carriers, such as MFS proteins being particularly enriched [11]. However, the phylogenetic relationships among these transporters remained largely unresolved. Our findings confirm the earlier suggestion by Schönknecht et al. (2013) that some *G. sulphuraria* transporters were acquired from non-eukaryotic lineages by HGT [12]. The MFS-SP subfamily, for instance, formed a polyphyletic group with a strong affinity to fungal sequences. Notably, some Galdieriales SP homologues grouped more closely with fungal sequences, implying multiple acquisition events and supporting the hypothesis that this gene family has undergone repeated HGT from fungal donors.

In contrast, the MFS-PHS subfamily showed a coherent monophyletic origin among Galdieriales species and clustered as a single, well-supported clade. While this group also exhibited phylogenetic proximity to fungal sequences, the fungal taxa involved differed from those associated with the SP family. This divergence suggests that the two MFS subfamilies may have been acquired independently, possibly reflecting different selective pressures or donor lineages. The lack of polyphyly within the MFS-PHS subfamily may indicate a single, earlier HGT event followed by vertical inheritance within Galdieriales [12].

A distinct pattern emerged for the APC family, which was only identified in Galdieriales and extremophilic prokaryotes. No homologues were detected in other eukaryotes, including closely related red algae, even under relaxed search thresholds. The APC clade formed a well-supported grouping with Archaea and some extremophilic Bacteria. This restricted taxonomic distribution suggests a gene transfer event from a prokaryotic donor into the *G. sulphuraria* lineage, potentially providing functional benefits for solute transport under acidic or metal-rich conditions [23].

The Nramp family revealed yet another pattern. Homologues of this divalent metal transporter were widespread, appearing across nearly all major prokaryotic and eukaryotic groups examined. Galdieriales species formed a distinct clade alongside those of *Cyanidioschyzon merolae* and *Cyanidiococcus yangmingshanensis*, with high bootstrap and SH-aLRT support. This group was a sister clade to Stramenopiles, suggesting a shared ancestry or retention of ancient homologues. Unlike the MFS subfamilies or the APC family, the Nramp transporters did not show evidence of recent or taxon-specific HGT but instead appear to reflect vertical inheritance within a widely distributed and conserved gene family.

Structural models of the Nramp family predicted by AlphaFold showed low pLDDT scores compared to those of the MFS and APC families (Figure S4), suggesting lower model confidence or potential structural divergence. This low structural confidence was consistent across *Galdieria* isolates, potentially indicating functional divergence of these transporters. The activity and structural conformation of Nramp transporters are profoundly influenced by the pH of their surrounding environment, which modulates both the electrochemical driving forces and the protonation states of key functional residues [24]. Under acidic conditions, the increased proton concentration directly affects the proton-coupled transport mechanism of Nramps. Structural and functional studies have shown that acidic pH favors the inward-facing or occluded conformations of Nramp proteins, likely as a means of facilitating substrate release into the cytoplasm following metal–proton symport [24,25]. This pH-dependent conformational bias is not only a structural phenomenon; it underpins the efficiency of the transport cycle by ensuring directionality and coordination between proton and metal ion flux. Understanding the pH-dependence of Nramp function is especially relevant in extremophiles. Beyond eukaryotic systems, pH modulation of Nramp function is particularly crucial in extremophilic organisms adapted to life in low-pH environments. Acidophilic microorganisms, such as *Acidithiobacillus ferrooxidans*, *Leptospirillum ferriphilum*, and *Ferroplasma acidarmanus*, inhabit ecological niches with pH values often below 3. In these species, metal ion uptake must occur against extreme proton gradients and in the presence of high extracellular proton concentrations, posing unique biophysical challenges to membrane transport processes [26]. To address these, *Nramp* homologs in acidophiles have evolved specialised adaptations to maintain structural integrity and functional efficiency under acidic stress. The low pLDDT scores in *Galdieriales* Nramp models could reflect a functional divergence and unique adaptations to acidic conditions or even flexible or disordered regions important for pH-sensing and transport. However, it is presumable that if these acidophile Nramps have unusual sequences or low homology, AlphaFold’s predictions become less confident.

Taken together, comparison across the three transporter families reveals contrasting evolutionary patterns. The MFS subfamilies (SP and PHS) both showed fungal affinities but differed in their degree of polyphyly and internal conservation. SP was found to be patchy and divergent, while *PHS* was seen as cohesive. The APC family is uniquely associated with extremophilic prokaryotes and is absent from all other eukaryotes surveyed, suggesting an isolated HGT event [23]. Meanwhile, the Nramp family stands apart as broadly conserved across taxa, showing no clear signs of recent HGT. These differences underline the mosaic origins of Galdieriales species transporter repertoire and suggest that adaptation to extreme environments was achieved through a combination of ancient inheritance and selective gene acquisition [27,28].

## 4. Materials and Methods

### 4.1. Selection of Target Genes

Protein-coding sequences were extracted in amino-acid format from *G. yellowstonensis* strain SAG 107.79 (NCBI BioProjectID PRJNA929444, BioSampleID SAMN33241403) were used to identify representatives from three major transporter families implicated in the biological processes of “Localization” (GO:0051179) and transport (GO:0006810): the Major Facilitator Superfamily (MFS), including Sugar Porter (SP) and Phosphate:H^+^ Symporter (*PHS*) families; the Amino Acid–Polyamine–Organocation (APC) family; and the Metal Ion Transporter Nramp family. Target genes were chosen based on transcriptomic data obtained previously, treating the species *G. yellowstonensis* with the rare earth element Cerium (25 mg/L) for one hour.

### 4.2. Intra-Genus Homolog Discovery

To assess the conservation and divergence of these proposed transcripts within Galdieriales, we conducted an intra-genus comparative analysis across five additional species from NCBI BioProjectID PRJNA929444: *Galdieria sulphuraria* ACUF 017 (BioSmpleID SAMN33241380), *Galdieria javensis* ACUF 074 (BioSmpleID SAMN33241404), *Galdieria daedala* ACUF 427 (BioSmpleID SAMN33241410), *Galdieria sulphuraria* ACUF 138 (BioSmpleID SAMN33241377), and *Galdieria partita* THAL 033 (BioSmpleID SAMN33241406). The amino-acid sequences from *Galdieria yellowstonensis* SAG 107.79 were used as queries in BLASTP from BLAST+ version 2.2.27 searches against local databases constructed from transcriptomic assemblies of these strains. Searches were conducted using BLAST+ v2.2.27 [29] with the following thresholds: e-value (*p*-value) ≤ 1 × 10^−6^, minimum identity ≥ 30%, and minimum query coverage ≥ 80%. BLASTP results were manually curated to retain only high-confidence homologs for each transporter family across the Galdieriales species.

### 4.3. Prediction of Transmembrane (TM) Domains

Identification and prediction of transmembrane (TM) domains were performed using web-based PROTTER v. 1.0 and Phobius softwares [30,31]. The borders of TM helices were also verified using the TMHMM web server 2.0 (https://services.healthtech.dtu.dk/services/TMHMM-2.0/; accessed on 10 April 2025).

### 4.4. Protein Structure Prediction

The three-dimensional (3D) structures of the target proteins were predicted using AlphaFold2, a deep learning–based tool for accurate protein structure prediction [32]. Protein sequences were submitted to the AlphaFold2 pipeline, and models were generated under default parameters. The confidence of the predicted structures was assessed using the predicted Local Distance Difference Test (pLDDT) scores, which provide per-residue estimates of model reliability. The predicted 3D protein structures were visualised and analysed using UCSF ChimeraX v. 1.9 [33].

### 4.5. Retrieval of Orthologs from Diverse Taxa

To contextualise Galdieriales transporter sequences within broader evolutionary lineages, each DEG sequence was also queried against the NCBI non-redundant (nr) protein database using BLASTP. Taxonomic filters were applied to retain representative hits from the following groups: Archaea (taxid:2157), Bacteria (taxid:2), Streptophyta (taxid:35493), Chlorophyta (taxid:3041), Rhodophyta (taxid:2763), Stramenopiles (taxid:33634), Alveolata (taxid:33630), Amoebozoa (taxid:554915), Fungi (taxid:4751), Choanoflagellates (taxid:28009), and Animals (taxid:33208). Searches used the same parameters described above (e-value ≤ 1 × 10^−6^, identity ≥ 30%, and coverage ≥ 80%). The top-scoring hits from each taxonomic group were retained to avoid overrepresentation bias.

### 4.6. Sequence Alignment and Curation

All selected sequences, both from Galdieriales and external taxa, were aligned using MAFFT v7.453 [34] with default settings. To improve the alignment quality for phylogenetic reconstruction, poorly aligned or divergent regions were pruned using Gblocks v0.91b [35]. Default parameters were used, except for the minimum block length, which was adjusted to 5 residues to allow for high conservation regions within transporter domains. Where sequence sets lacked sufficient diversity or suffered from short alignable regions, manual curation was applied to remove problematic sequences.

### 4.7. Phylogenetic Tree Reconstruction

Phylogenetic relationships were inferred using IQ-TREE v2.0.3 [36]. To reduce phylogenetic bias, a multi-step filtering was applied before phylogenetic inference, using CD-HIT v4.8.1 with a 90% identity threshold [37], pairwise identity assessment, calculating an all-vs.-all pairwise identity matrix using Clustal Omega v.1.2.4. A validation step was then performed, generating a BLASTp database from the final alignment using makeblastdb, followed by an all-vs.-all BLASTp search. For each alignment, the best-fit amino acid substitution model was automatically selected using the inbuilt -m TEST function, which tests multiple candidate models using the Bayesian Information Criterion (BIC). Maximum likelihood (ML) trees were reconstructed with the following statistical supports: 10,000 ultrafast bootstrap replicates (UFBoot) [38]; 1000 SH-like approximate likelihood ratio test (SH-aLRT) replicates [39]. Nodes were considered robust when support values exceeded 95% (UFBoot) and 90% (SH-aLRT). The HGTphyloDetect pipeline [40] was used to confirm HGT events involving Galdieriales species.

## 5. Conclusions

The role of HGT in shaping the Galdieriales genomic landscape is now well established. The present findings further illustrate that this process likely extended to key transporter genes involved in metal uptake, solute movement, and homeostasis as functions critical to survival in geothermal niches. While the current study did not assess gene expression changes in response to metal stress per se, the overrepresentation of these transporter families in the transcriptome and their phylogenetic placements support a model in which horizontal acquisition complemented ancient gene lineages to enhance environmental tolerance in this extremophilic red alga.

## Data Availability

The original contributions presented in this study are included in the article/Supplementary Materials. Further inquiries can be directed to the corresponding author(s).

## Supplementary Materials

The following supporting information can be downloaded at https://www.mdpi.com/article/10.3390/plants14132043/s1. Additional analyses supporting the findings of this study are included in the Supplementary Materials.
