# Supplementary material for: Mosaic Evolution of Membrane Transporters in Galdieriales
Source: Plants (Basel). 2025 Jul 3;14(13):2043. doi: 10.3390/plants14132043 (PMC12252169; doi:10.3390/plants14132043)
Supplement: Supplementary file 1 [file plants-14-02043-s001.zip › FigureS2.pdf]

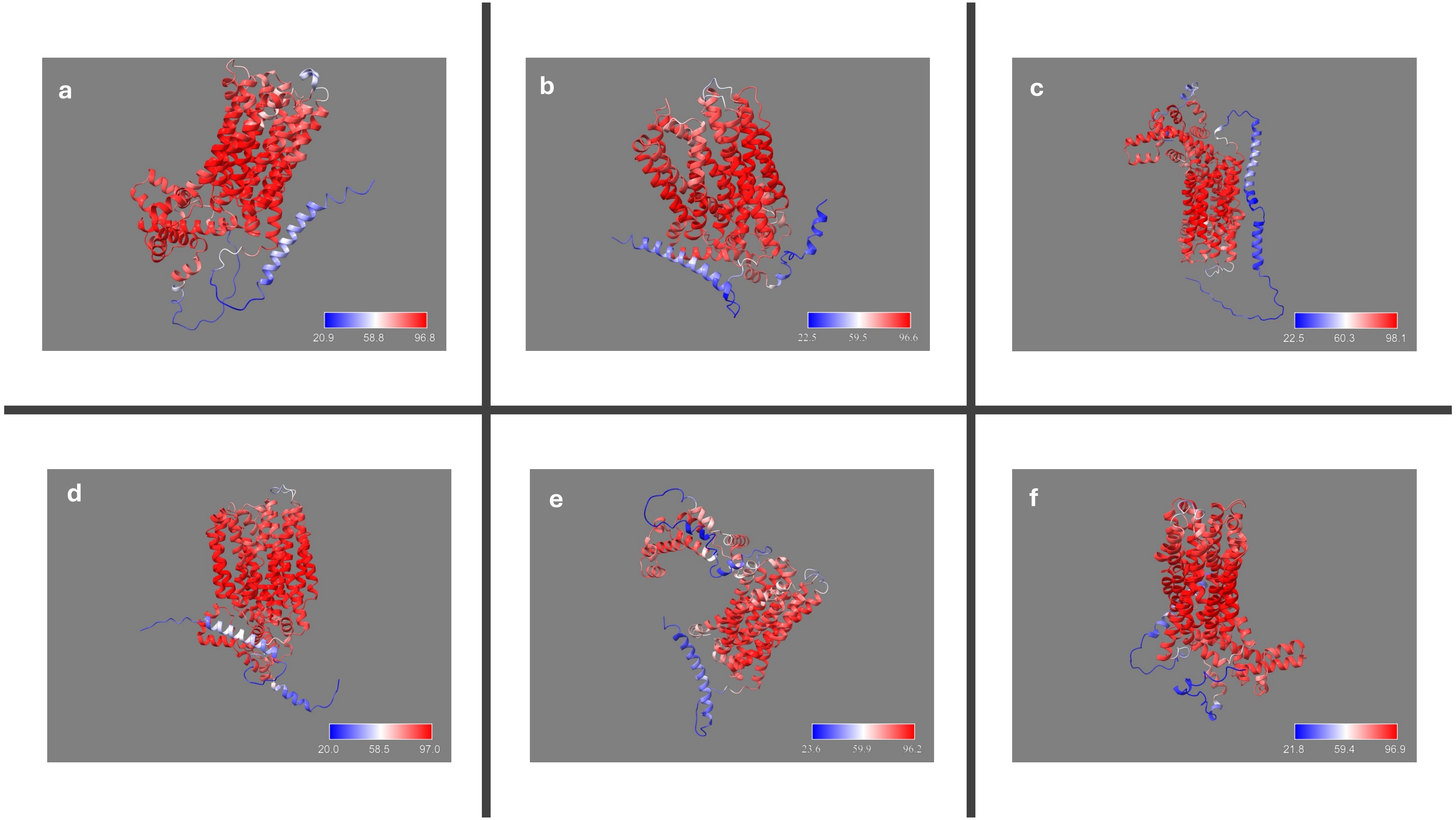

Figure S2. AlphaFold modelling of the Major Facilitator Superfamily (MFS), Phosphate:H$^+$ Symporter (PHS) family; **a)** Predicted Alphafold structure for the MFS_PHS from *G. sulphuraria*, ACUF 017; **b)** Predicted Alphafold structure for the MFS_PHS from *G. javensis*, ACUF 074; **c)** Predicted Alphafold structure for the MFS_PHS from *G. sulphuraria*, ACUF 138; **d)** Predicted Alphafold structure for the MFS_PHS from *G. daedala*, ACUF 427; **e)** Predicted Alphafold structure for the MFS_PHS from *G. yellowstonensis*, SAG107.79; **f)** Predicted Alphafold structure for the MFS_PHS from *G. partita*, THAL033.
